# Supplementary material for: Global sensitivity analysis of a dynamic model for gene expression in Drosophila embryos
Source: PeerJ. 2015 Jun 18;3:e1022. doi: 10.7717/peerj.1022 (PMC4476099; doi:10.7717/peerj.1022)
Supplement: Supplemental Information 2 [file peerj-03-1022-s002.pdf]

Noname manuscript No.
(will be inserted by the editor)

Supplemental Information: Global sensitivity analysis of a dynamic model for gene expression in *Drosophila* embryos

Gregory D. McCarthy · Robert A. Drewell · Jacqueline M. Dresch

the date of receipt and acceptance should be inserted later

Gregory D. McCarthy
Hampshire College, 893 West St, Amherst, MA 01002
E-mail: gm13@hampshire.edu

Robert A. Drewell
Biology Department
Clark University, Worcester, MA 01610
E-mail: rdrewell@clarku.edu

Jacqueline M. Dresch
Department of Mathematics
Amherst College, Amherst, MA 01002
E-mail: jdresch@amherst.edu

A.

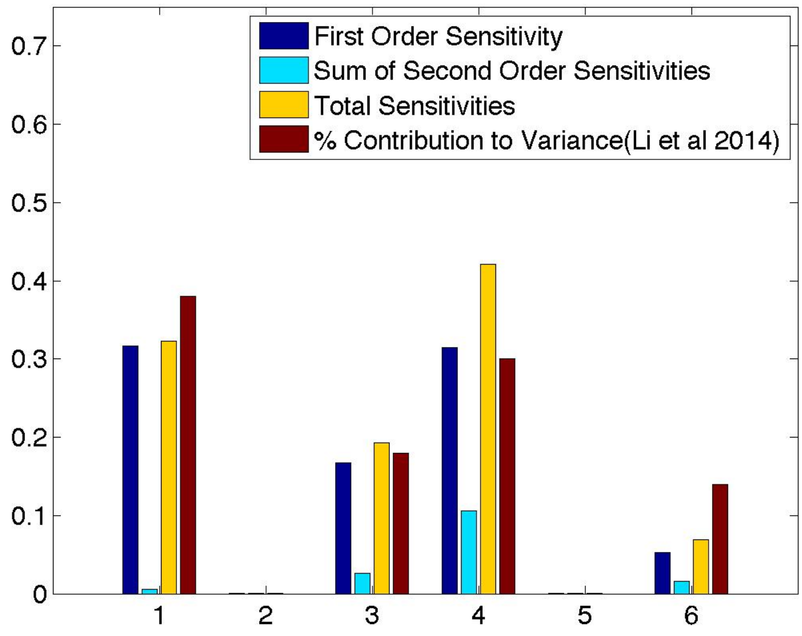

B.

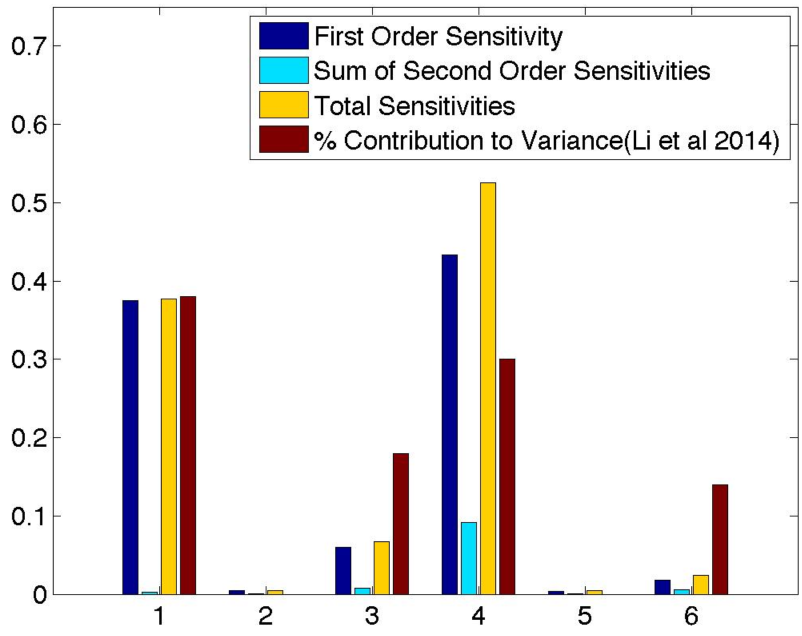

Fig. S1. Qualitative similarities between parameter sensitivities at the most anterior nucleus and experimental measurements. A. Ubiquitous gene with initial concentrations of 1.0; First and second-order sensitivities at the most anterior nucleus at $t = 4$ minutes. B. Anterior maternally deposited gene; First and second-order sensitivities at the most anterior nucleus at $t = 2$ minutes. In both panels, along the x-axis are the parameters corresponding to: 1. Transcription, 2. mRNA Diffusion, 3. mRNA Decay, 4. Translation, 5. Protein Diffusion, and 6. Protein Decay.

A.

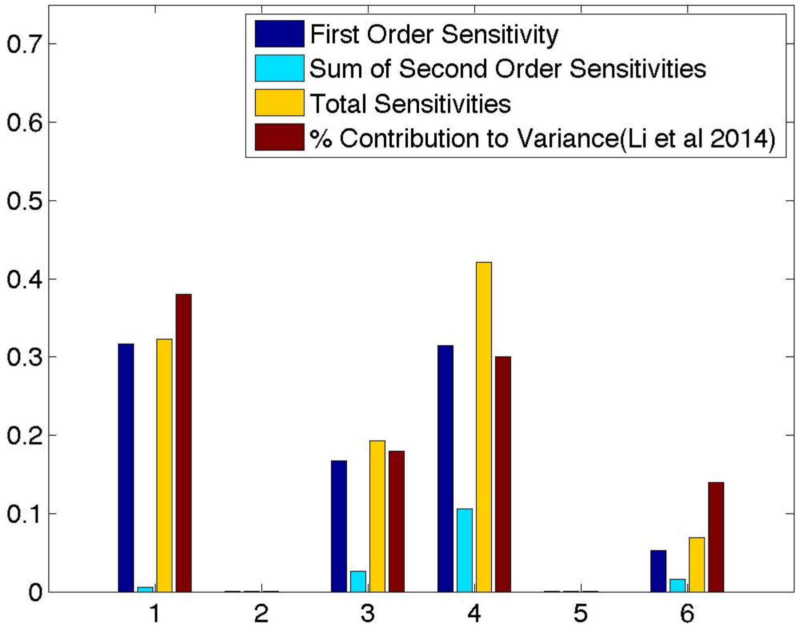

B.

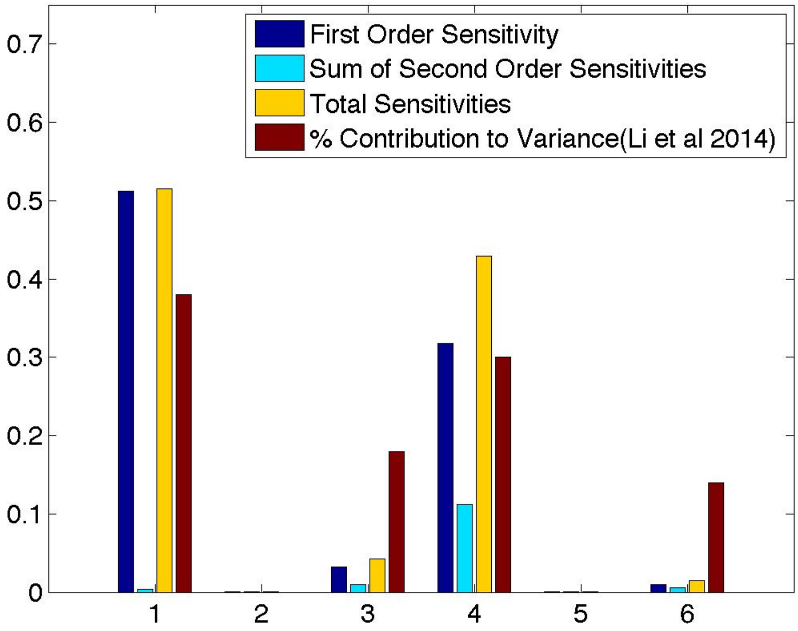

Fig. S2. Qualitative similarities between parameter sensitivities at the most posterior nucleus and experimental measurements. A. Ubiquitous gene with initial concentrations of 1.0; First and second-order sensitivities at the most posterior nucleus at $t = 4$ minutes. B. Anterior maternally deposited gene; First and second-order sensitivities at the most posterior nucleus at $t = 2$ minutes. In both panels, along the x-axis are the parameters corresponding to: 1. Transcription, 2. mRNA Diffusion, 3. mRNA Decay, 4. Translation, 5. Protein Diffusion, and 6. Protein Decay.

A.

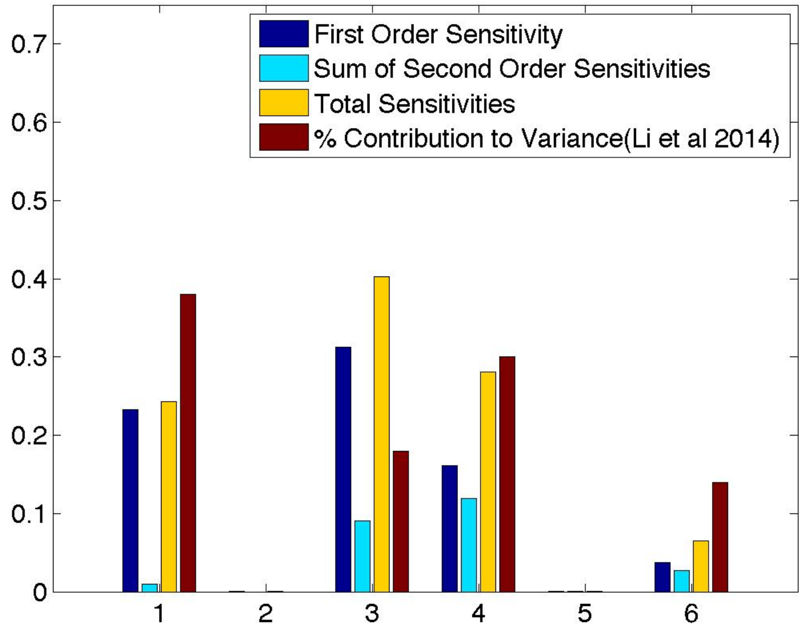

B.

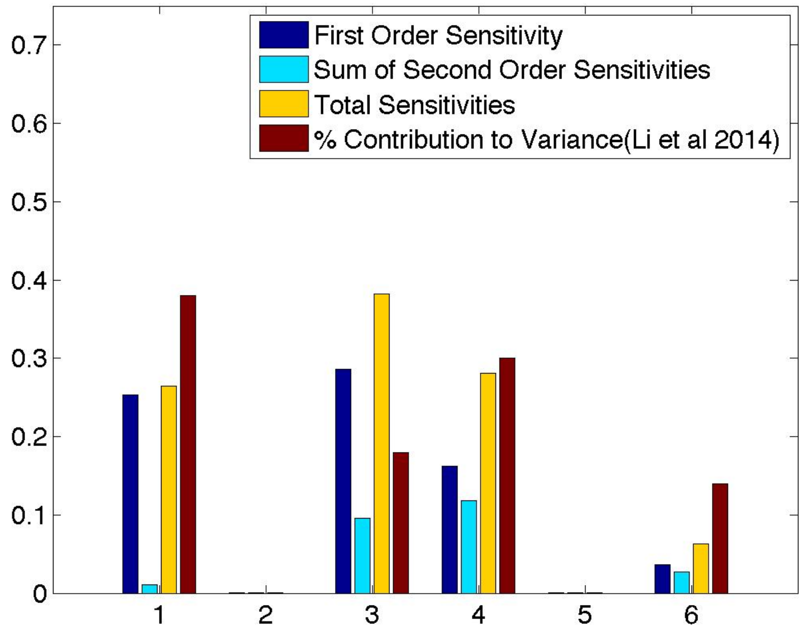

Fig. S3. Comparison of parameter sensitivities at the most anterior nucleus to experimental measurements at a later time point. A. Ubiquitous gene with initial concentrations of 1.0; First and second-order sensitivities at the most anterior nucleus at $t = 10$ minutes. B. Anterior maternally deposited gene; First and second-order sensitivities at the most anterior nucleus at $t = 10$ minutes. In both panels, along the x-axis are the parameters corresponding to: 1. Transcription, 2. mRNA Diffusion, 3. mRNA Decay, 4. Translation, 5. Protein Diffusion, and 6. Protein Decay.

A.

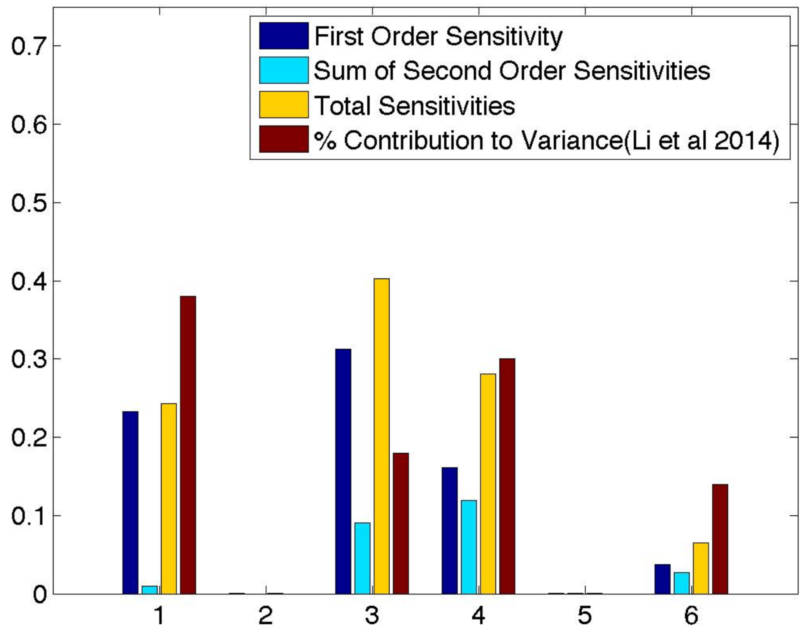

B.

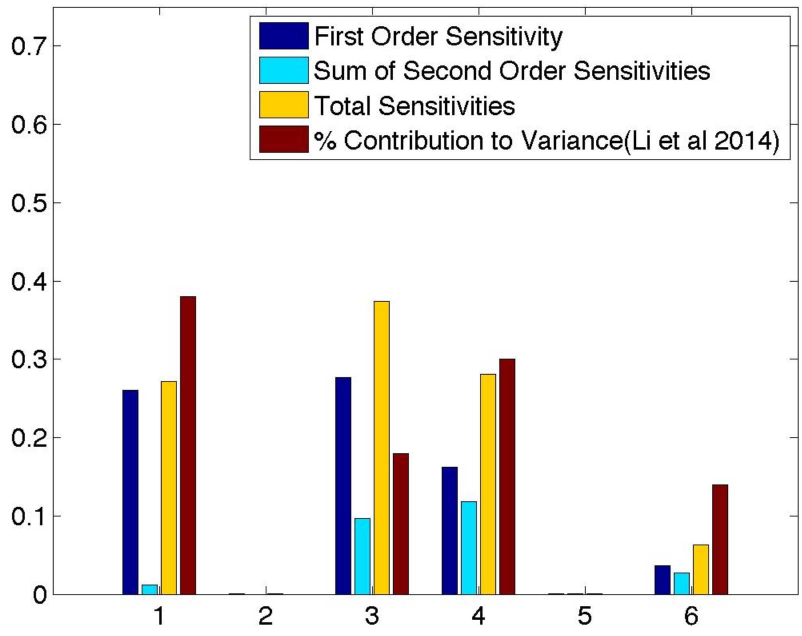

Fig. S4. Comparison of parameter sensitivities at the most posterior nucleus to experimental measurements at a later time point. A. Ubiquitous gene with initial concentrations of 1.0; First and second-order sensitivities at the most posterior nucleus at $t = 10$ minutes. B. Anterior maternally deposited gene; First and second-order sensitivities at the most posterior nucleus at $t = 10$ minutes. In both panels, along the x-axis are the parameters corresponding to: 1. Transcription, 2. mRNA Diffusion, 3. mRNA Decay, 4. Translation, 5. Protein Diffusion, and 6. Protein Decay.

A.

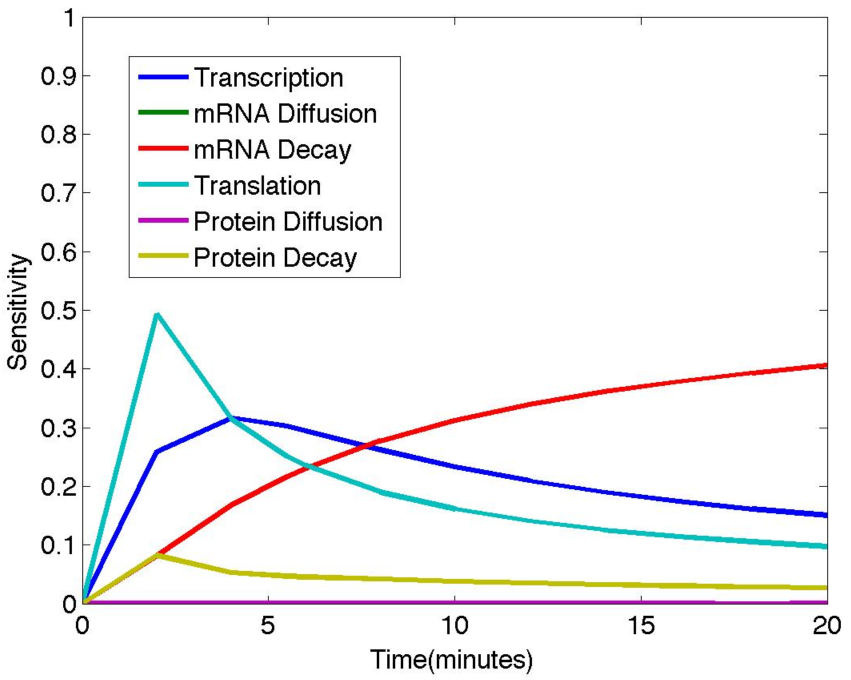

B.

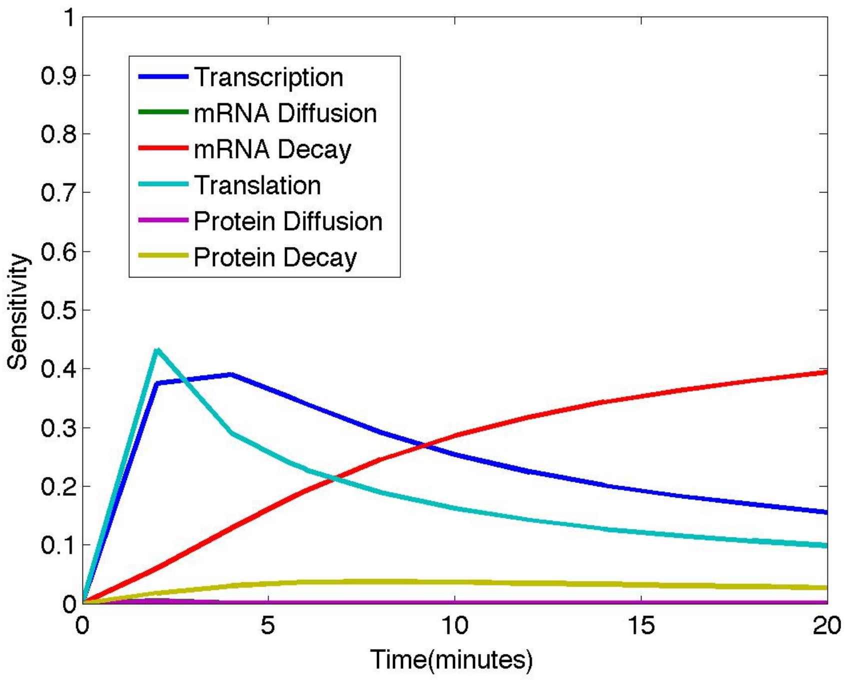

Fig. S5. Temporal dynamics of parameter sensitivities at the most anterior nucleus. A. First-order parameter sensitivities at the most anterior nucleus over time for a ubiquitous gene with initial concentrations of 1.0. B. First-order parameter sensitivities at the most anterior nucleus over time for an anterior maternally deposited gene

A.

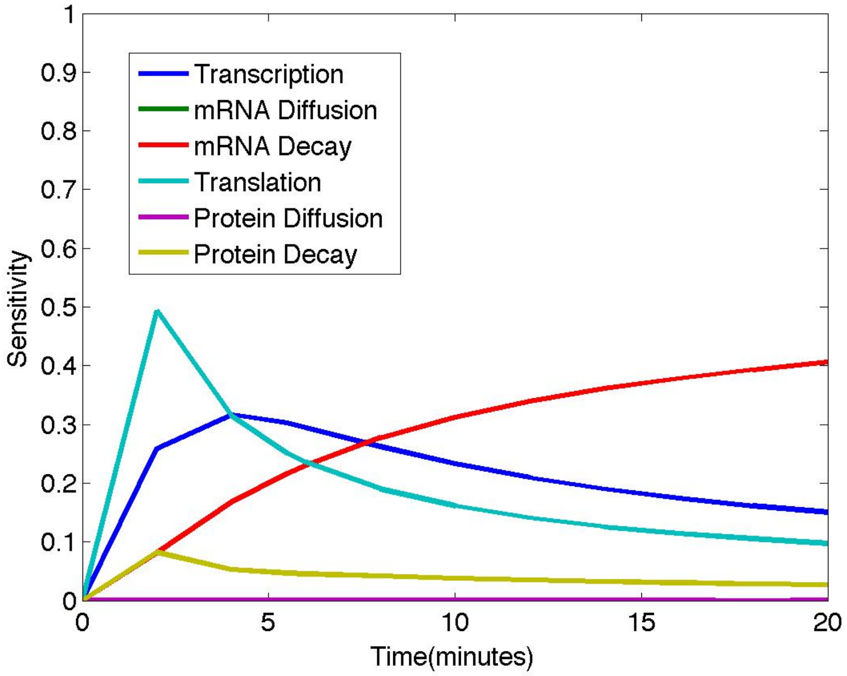

B.

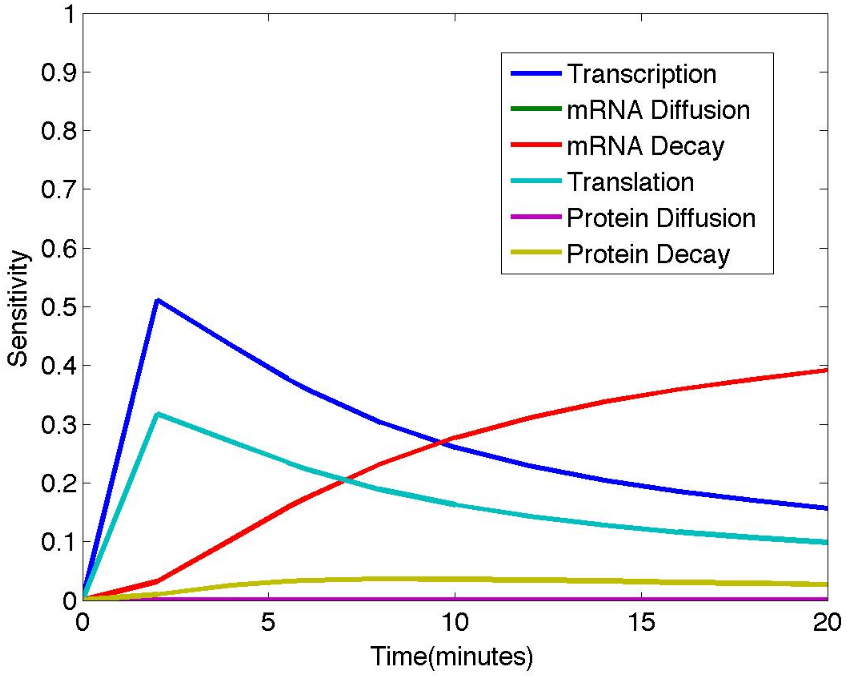

Fig. S6. Temporal dynamics of parameter sensitivities at the most posterior nucleus. A. First-order parameter sensitivities at the most posterior nucleus over time for a ubiquitous gene with initial concentrations of 1.0. B. First-order parameter sensitivities at the most posterior nucleus over time for an anterior maternally deposited gene
